# A New Era in Salivary Gland Carcinoma Treatment: A Case Report

**DOI:** 10.7759/cureus.42983

**Published:** 2023-08-05

**Authors:** Joana C Mendonça, Ana Barbosa, Claudia Vieira, José Dinis

**Affiliations:** 1 Oncology, Hospital da Senhora da Oliveira, Guimarães, PRT; 2 Medical Oncology, Instituto Português de Oncologia do Porto, Porto, PRT; 3 Oncology, Instituto Português de Oncologia do Porto, Porto, PRT

**Keywords:** her2-positive, local treatment, actionable genomic alterations, next generation sequencing (ngs), salivary gland carcinoma

## Abstract

Salivary gland cancers are rare and heterogenous malignancies which makes it hard to standardize treatments with good evidence levels. The localized disease approach is well established, with surgery to the primary site and adjuvant radiation therapy in patients with high-risk features. Treatment of advanced disease should be multidisciplinary. Local approaches, which include radiation therapy, surgery, and thermoablation, among others, have the potential to achieve durable disease control with low toxicity. Chemotherapy has shown disappointing results, so systemic treatment should be guided by actionable genetic alterations, which in salivary gland cancers rely on the histologic type. When directed molecular tests are not useful, a multigene panel should be performed. This case is a good example of how to integrate all these possible tretaments in clinical practice, including molecular testing and target treatment.

## Introduction

Salivary gland carcinomas (SGCs) are rare malignancies representing 1-5% of all head and neck cancers (HNC), with an incidence of 0.69 cases per 100,000 people per year worldwide [1,2]. They are a heterogeneous group of cancers with 24 SGC types described in the World Health Organization classification system [3]. Histologic subtypes have different behavior and actionable genomic alterations (AGA), such as human epidermal growth factor receptor 2 (HER2) amplification. Hence, choosing the best treatment is hard and evidence comes from small patient series, case reports, and, more recently, basket trials [1,2,4].

In metastatic and recurrent disease, a multidisciplinary approach with locoregional and systemic treatments plus molecular testing to detect AGA is of major importance and may improve patient survival and quality of life [1,2]. Here, we describe a case of a mucoepidermoid salivary gland carcinoma with long survival and disease control with locoregional treatment and target therapies strategies.

## Case presentation

A 57-year-old man with a medical history of dyslipidemia was noted to have a right cervical nodule in January 2016. An ultrasonography and core biopsy diagnosed it as a high-grade mucoepidermoid salivary gland carcinoma (ME-SGC). In March 2016, he underwent excision of the right submandibular salivary gland and suprahyoid lymph node dissection, staged as pT1N0Mx with a close free margin (less than 1 mm), followed by local adjuvant radiation therapy (RT).

In October 2016, he was diagnosed with a right cervical lymph node recurrence and underwent right-side cervical dissection, with three out of eight nodes metastasized and with focal extranodal invasion, followed by chemoradiotherapy (CRT) with two cycles of cisplatin 100 mg/m^2^, with no relevant toxicity or morbidity.

In December 2017, two pulmonary nodes were noted in the left inferior lobe, which biopsy confirmed a ME-SGC recurrence, and a next-generation sequencing (NGS) test showed an ERB2 amplification and low tumor mutational burden (TMB), with three mutations/MB. A fluorodeoxyglucose-positron emission tomography-computerized tomography (FDG-PET-CT) excluded other metastatic sites and the patient was submitted to stereotaxic body RT (SBRT) to the lung lesions on February 2018. One month later, a new lung recurrence was detected in the left inferior lobe, with the stability of the previous sites, and SBRT was performed on this new nodule.

In July 2018, he had disease progression in both lungs and was treated with three cycles of systemic treatment docetaxel and trastuzumab with good partial response in the FDG-PET-CT that showed only one lesion in the left lung for which he had metastasectomy.

In July 2019, a new pulmonary bilateral progression was seen and managed with SBRT. Six months later a hepatic nodule was detected and submitted to metastasectomy. Pathological evaluation confirmed a ME-SGC recurrence, with HER2 positive (3+) by immunohistochemistry. A new NGS test showed ERB2 amplification and a high TMB of 13 mutations/MB.

**Figure 1:**
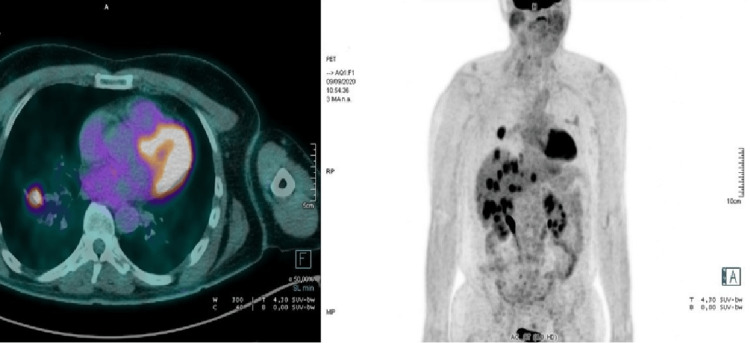
FDG-PET-CT image from September 2020. Right: full body FDG-PET-CT image from September 2020 showing lesions with FDG uptake in the lung and liver. Left: a close-up of the pulmonary lesion with high FDG uptake. FDG-PET-CT: fluorodeoxyglucose-positron emission tomography-computerized tomography

**Figure 2:**
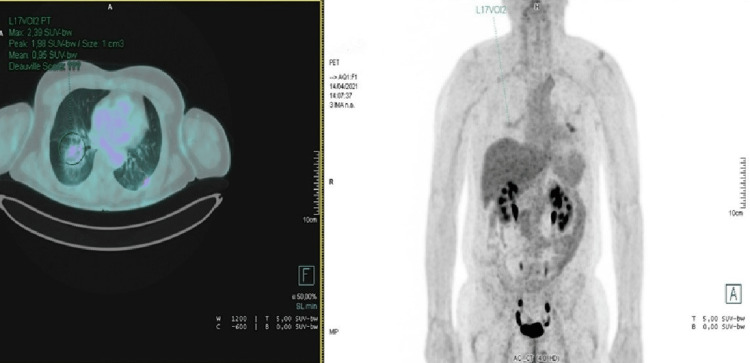
FDG-PET-CT image from April 2021. Right: full body FDG-PET-CT image from April 2021 showing full resolution of previous metastatic lesions. Left: a close-up of the thorax, with no FDG uptake on the right pulmonary lesion. FDG-PET-CT: fluorodeoxyglucose-positron emission tomography-computerized tomography

In September 2020, he progressed with pulmonary and hepatic metastasis (Figure 1) and restarted treatment with docetaxel and trastuzumab with a resolution of all lesions (Figure 2).

However, after eight cycles, pulmonary progression was detected and systemic treatment was changed to trastuzumab emtansine (TDM-1) in September 2021, continued for 16 cycles, with stable disease, until new lung progression in October 2022.

The patient maintained a good performance status throughout his disease course and has now enrolled in a phase I study designed for patients with HER2-positive metastatic tumors, in which he is still enrolled at this date.

## Discussion

In the localized disease setting, surgical excision of the affected salivary gland is the standard of care. Adjuvant RT is warranted in patients with high-grade tumors, positive or close margins, perineural or lymphovascular invasion, lymph node metastases, and T3-4 tumors [1,2]. In the submandibular gland, the risk of micrometastasis to the neck is 33%, and adjuvant RT, when recommended, should include the neck region [1].

In SGC the most frequent metastatic site is the lung. Follow-up imaging after initial treatment of localized disease should be directed to the primary site and thorax for the first two years and guided by symptoms and physical examination thereafter [1].

In HNC oligometastatic disease, local treatment of metastatic sites is not well established, however, several studies have shown longer systemic disease control with good quality of life [4,5]. In SGC there is less evidence in this concern. In a retrospective study of 109 patients with acinic cell carcinomas who underwent pulmonary metastasectomy, the authors recommend this approach only if complete resection is feasible and the relapse occurs more than 36 months after initial treatment [6]. In high-grade ME-SGC there aren't any series showing the benefit of metastasis local treatment, however, in our patient, this strategy allowed some disease control for several years, in a metastatic cancer with very few systemic treatment options [1,2].

Systemic therapy has limited efficacy in SGC. Small prospective series and retrospective studies have shown that some AGA has a high prevalence in specific SGC histologic subtypes. Several guidelines recommend that patients should be offered molecular alterations testing according to histology - NTRK gene fusion in secretory carcinoma, HER2 and androgen receptor status in adenocarcinoma and salivary gland carcinoma. For histologies known to have low AGA or in patients with good performance status and without therapeutic options, a multigene panel should be offered, like NGS [1,2,7]. NGS testing allows the detection of AGA but also grouping patterns of mutations into mutational signatures, like homologous recombination deficiency, TMB, and microsatellite instability [7]. In our patient, the first NGS showed an ERB2 amplification and low TMB, and the second had a high TMB which may allow, for example, pembrolizumab treatment through agnostic approval from the phase II trial KEYNOTE-158 [8]. Our case also shows the importance of repeating molecular profiles in progression biopsies, because of the tumor heterogeneity, but also the systemic treatment pressure in the tumor cells and environment may change its profile throughout the disease course.

In high-grade ME-SGC, EGFR is overexpressed in 72.7% and ERB2 is amplified in 8.3%. The latter, although less frequent, is amenable to HER2 target therapy [9]. Several prospective studies treating HER2-amplified SGC with trastuzumab plus chemotherapy or TDM-1 included patients with ME-SGC showed a durable response in this specific histology [10-13]. In our patient, the longer response registered was with HER2 target therapy with a progression-free survival of 10 months with trastuzumab plus docetaxel and 13 months with TDM-1.

## Conclusions

This case is a good example of how multidisciplinary management is mandatory in the SGC advanced setting to achieve better survival and quality of life. Also represents the urgency to standardize AGA testing in the SGC and include them in treatment algorithms, as suggested by recent guidelines. Inclusion in clinical trials is always an option and should be encouraged in rarer tumors, like SGC.
